# Patient Trust and Reputation Management in Dental and Ophthalmology Practices

**DOI:** 10.22336/rjo.2025.08

**Published:** 2025

**Authors:** Cristina Stanciu (Neculau), Aida Geamănu, Roxana Monica Purcărea

**Affiliations:** 1 Department of Marketing and Medical Technology, “Carol Davila” University of Medicine and Pharmacy, Bucharest, Romania; 2 Department of Ophthalmology, “Carol Davila” University of Medicine and Pharmacy, Bucharest, Romania; 3 “Dr. Carol Davila” Clinical Hospital of Nephrology, Bucharest, Romania

**Keywords:** reputation management, patient trust, dental practices, ophthalmology practices

## Abstract

Reputation is one of the most significant aspects of an organization. It plays a crucial role in Dentistry and Ophthalmology, significantly influencing the relationship between doctors and patients. Reputation management in these two fields is viewed as a strategic process through which dentists and ophthalmologists can establish patients’ trust, thereby ensuring high-quality services and patient satisfaction. The study conducted in this paper aimed to analyze patients’ opinions on how the reputation of medical offices and trust in ophthalmologists and dentists influence their decision to choose a particular office. The results showed that the doctors’ professionalism and the quality of the equipment used are the most critical factors influencing patients’ trust in ophthalmology and dental offices. Additionally, patients primarily evaluate the reputation of these practices based on their previous experiences or recommendations from family and friends.

## Introduction

Many management specialists [1] have recently studied a company’s reputation. The emergence and development of new disruptive technologies [2,3] have increased their importance within organisations, which has determined enterprises to be more attentive to their market image [4].

Examining the evolution of the “corporate reputation” concept, it is worth noting that it emerged in 1970. It is seen as an essential element that can influence activity conducted at the organisational level [5].

Reputation management is regarded as the management behaviour that has the role of identifying how external public can perceive a company from the perspective of what it is capable of accomplishing in a specific time, of what it is struggling to become, but also from the point of view of the impact it has at the societal level in general [6]. Some authors believe that this reflects the reputation or esteem of an organisation, being seen as a result of the most critical organisational attributes and its past actions [7]. Reputation is an intangible asset for both clients and organisations. Often, consumers tend to orient towards companies with a higher reputation, thus preferring reputable companies with a well-established name in the market [8].

Some authors view reputation as a competitive advantage in enterprises that should be continually invested in to enhance their market position [9]. Employees [10], investors, clients (through their reviews), the marketing activities conducted at the organisational level, and all their other actions collectively enhance a company’s attractiveness [11].

From a medical field’s perspective, it is essential to note that branding and reputation management are crucial elements in building and maintaining the image, identity, and credibility of enterprises and professionals operating in this field [12]. A doctor’s reputation, regardless of the specialty in dentistry or ophthalmology, is gradually built through patients’ experiences and their recommendations to family or friends. Additionally, the doctor’s or medical office’s online and offline presence plays a crucial role in creating a reputation [13]. Social media presence represents another method that enables medical specialists to interact with patients, promoting themselves and their services within medical offices [14].

A strong reputation attracts many patients, increases doctors’ notoriety [15], and retains existing clients while also building and nurturing a strong market image. The competencies of the medical specialists who conduct their activity within them, their attitude towards patients, and the medical equipment utilized play an essential role in reinforcing a medical office’s reputation. Moreover, transparency and the communication process [16] can positively influence a medical office’s reputation during a crisis.

In both Dentistry and Ophthalmology, a series of factors can impact patients’ trust in the doctors who consult them. Therefore, the first aspect refers to the professional quality of the medical specialists. Their experience, professionalism, and reputation [17,18] are among the most significant factors influencing patients’ decisions to choose a specific medical office. Creating a brand in this field automatically increases reputation, trust [19], and patients’ loyalty [20].

On the other hand, patients place high importance on how doctors communicate with them, as well as on how all information regarding their conditions is transmitted. Patients’ recommendations or reviews in the online environment contribute to the reputational increase within the two fields [21,22]. They play a crucial role in establishing trust between potential patients and doctors, as well as in their medical offices. Another essential element in increasing the trust [23] that patients assign to doctors and medical offices is the hygiene conditions within health facilities. The more they perceive medical care in the best hygienic conditions, the greater the trust in the respective medical services. Thus, their reputation is much better.

In Dentistry and Ophthalmology, medical specialists must make a concerted effort to enhance the reputation of the medical offices in which they work. They must be present at the online environment level [24] to provide patients with all the necessary information and to discuss their conditions with them. Additionally, doctors have to receive permanent feedback from patients to be able to observe their opinion regarding their image [25], how they perceive the provided services, but also the aspects that should be improved within the medical offices so that the level of satisfaction felt to be as high as possible [26]. A trustworthy relationship between medical specialists and patients is crucial for delivering high-quality, patient-centered medical care [27]. Another aspect that doctors should strive to achieve to maintain a high level of trust in themselves [28] and in the medical offices they operate is to invest in their education. Because of this, they need to improve themselves continually, develop professionally [29], and stay up to date with all the new developments in their field of work.

Considering all the aspects mentioned above, it is worth noting that reputation and trust are the most essential pillars in Dentistry and Ophthalmology. Effective management in these fields contributes to attracting a large number of patients and to the loyalty of existing ones. To satisfy patients [30] and help them enjoy success, doctors should emphasize these two aspects and the factors that can influence them.

## Materials and methods

Taking into consideration the importance that patients give both to the reputation of medical offices and the doctors who work within them, we considered necessary to conduct a quantitative study in which we observed the opinion of patients regarding how the reputation of medical offices and the trust they have in ophthalmologists and dentists influence the decision to choose a particular medical practice. For this purpose, an online survey was conducted between September and December 2024 among patients who had visited an ophthalmology or dental office within the last 3 years. The research was performed on a sample of 152 people. To collect the information, a questionnaire was created, posted online, and later distributed to the respondents. The first two questions served as a filter, selecting only individuals who are part of the researched collective, specifically patients who have attended both a dental and an ophthalmological consultation within the last three years.

Analyzing the respondents from the perspective of their demographic profile, it was observed that the majority of respondents were women (57%), while 43% were men. The survey participants were divided into three age groups: 39% were aged between 36 and 45 years, 28% were between 46 and 55 years old, and 21% of the respondents were over 55 years old. In contrast, only 12% of the participants were between 25 and 35 years old.

From the perspective of their residential environment, 88% of them lived in urban areas, while 12% lived in rural areas. Sixty-one percent of those who participated in the study completed their bachelor’s degrees, 37% pursued master’s degrees, while only 3% obtained a PhD. From the perspective of the monthly net income collected by the respondents, 41% of them had a monthly income between 4,501 and 5,500 lei, 34% earned between 5,501 and 6,500 lei, and 14% earned between 3,500 and 4,500 lei. Only 11% of the study participants had a net monthly income of over 6,500 lei.

**Table 1:** The demographic profile of the respondents

Respondents’ sex	Frequency	Percent (%)
Men	66	43%
Women	86	57%
**Total**	**152**	**100.00%**
		
**Respondents’ age**		
25-35 years old	18	12%
36-45 years old	59	39%
46-55 years old	43	28%
Over 55 years old	32	21%
**Total**	**152**	**100%**
		
**Respondents’ residence environment**		
Rural	18	12%
Urban	134	88%
**Total**	**152**	**100%**
		
**Last school completed by respondents**		
Bachelor	92	61%
Master	56	37%
PhD	4	3%
**Total**	**152**	**100%**
		
**Respondents’ monthly net income**		
3500-4500 lei	22	14%
4501-5500 lei	63	41%
5501-6500 lei	51	34%
Over 6500 lei	16	11%
**Total**	**152**	**100%**

## Results

Regarding the results obtained, the respondents specified that the reputation of the medical office plays a vital role in the decision-making process. In the case of ophthalmology medical offices, the average obtained was 3.9, while in the case of dental offices, the average was 4.1, indicating that patients place a higher importance on reputation when choosing a dental office. On the other hand, the respondents specified that the reputation of the ophthalmologist (Average obtained: 4.3) or the dentist (Average obtained: 4.4) plays a significant role in this decision. It was thus observed that patients gave a special importance to the doctor with whom they came into contact, the average obtained being higher than that recorded at the level of medical offices. For them, the person who consulted and treated them was very important, whereas the unit where the medical services were provided was less so.

**Table 2:** The importance of medical offices and doctors’ reputation within the decisional process

Criterion	Average obtained
Reputation of the ophthalmology medical office	3.9
Reputation of the dental office	4.1
Reputation of the ophthalmologist	4.3
Reputation of the dentist	4.4

Regarding the sources of information that patients use to evaluate the reputation of an ophthalmology or dental office, it was observed that they considered their previous experience. In the case of ophthalmology, the respondents specified that the primary sources of information they use are online reviews (14%) or the medical office’s presence in the online environment (18%). Comparatively, it was observed that the weight obtained for these sources of information was lower in the case of dental ones: online reviews (9%) and the online presence of the medical office (8%). Additionally, patients believed that online sources were more critical when evaluating the reputation of eye clinics than dentists, and they preferred to obtain information from friends or family members.

**Table 3:** The primary sources of information used by patients to evaluate the reputation of ophthalmology and dentistry medical offices

	Percent (%)	Percent (%)
	Ophthalmology medical office	Dentistry medical office
Friends/family recommendations	22%	31%
Online reviews	14%	9%
The medical office’s online presence	18%	8%
The doctor’s online presence	8%	9%
Previous experience	38%	43%
**Total**	**100%**	**100%**

Regarding the respondents’ opinions on the most critical aspects that can increase their trust in ophthalmological and dental offices, it was observed that, in both cases, patients considered the doctor’s professionalism and the medical equipment used to be the most critical elements. In the case of dental offices, patients believed that the hygiene conditions and the kindness of the auxiliary staff contributed significantly to building trust. In contrast, in the case of ophthalmology, the communication between doctor and patient was considered to have a more critical role in building trust.

**Table 4:** The main factors that influence the respondents’ trust level in ophthalmology and dentistry medical offices

	Percent (%)	Percent (%)
	Ophtalmology medical office	Dentistry medical office
Doctor and patient communication	18%	15%
Doctor’s professionalism	37%	40%
Hygienic conditions	9%	13%
Kindness of the medical staff	3%	6%
Utilized equipment	33%	26%
**Total**	**100%**	**100%**

Regarding how the prices charged can influence patients’ trust in medical offices, it was observed that, in both cases, they agreed that their trust was often influenced by the costs charged at the level of health units. 62% of the respondents affirmed that the prices in eye doctor’s offices influenced their trust in that health facility. In comparison, 78% affirmed that those in dental offices could affect their trust in such health facilities.

61% of those who participated in the research would recommend an ophthalmology practice to friends or relatives based on its online reputation, without having had direct experience. In comparison, 41% of them mentioned that they would be willing to do this in the case of medical dental practices. It was also observed at that point that, in the case of dental medical offices, the direct experience and contact that patients had with the dentist played a crucial role, unlike in ophthalmology, where reputation and visibility in the online environment were much more critical.

## Discussion

Patient trust is one of the most critical factors in the success and development of ophthalmology and dental practices. Their reputation is often the result of the professionalism of the doctors, the communication process with patients, the transparency of their services, and their level of visibility in the online environment. In an increasingly competitive environment, where patients have the opportunity to get information from multiple sources about the medical practices existing on the market, their perception of the professionalism of the doctors, the quality of the services provided and also the reliability have an essential role in the level of the decision-making process of choosing a particular cabinet.

This study aimed to analyze how the reputation of medical offices influences patients’ trust in ophthalmologists and dentists, affecting their decision to choose a particular office. The survey was conducted between September and December 2024 on a sample of 152 respondents. They have accessed both ophthalmological and dental services in the last three years. Data were collected using an online distributed questionnaire, and participant selection was based on filter questions to include only patients relevant to the study.

The results illustrated that patients give more importance to the doctor’s reputation than the practice’s, indicating that trust is closely related to direct experience with the specialist. In the case of dental offices, reputation plays a more critical role (Average 4.1) than in the case of ophthalmology (Average 3.9). Still, the doctor’s reputation has a more substantial influence (Average 4.3 - ophthalmology, Average 4.4 - dentistry). Regarding sources of information, previous experience was considered the most important for both eye and dental practices. However, patients tend to evaluate the reputation of ophthalmologists more often through online reviews and digital presence, whereas personal recommendations carry more weight in dentistry.

Additionally, the factors contributing to increased patient trust vary between the two specializations. The professionalism of the doctor and the equipment used are the most important in both fields. However, in ophthalmology, doctor-patient communication is essential, whereas in dentistry, hygiene and the friendliness of the staff have a more significant influence. The study also highlighted the impact of prices on patient trust: 62% of respondents believed that the rates charged in ophthalmology influence their confidence in the office, the percentage being even higher (78%) in the case of dental offices. In addition, online reputation has a more significant impact in ophthalmology, with 61% of patients willing to recommend a practice based solely on it, compared to 41% in dentistry, where direct experience plays a much more critical role.

Although the presented study can illustrate the impact of patients’ reputation and trust on the choice of dental and ophthalmology practices, it is worth noting that, at its current level, there are also some limitations. The first limitation refers to the small sample size (152 respondents), which did not allow us to extrapolate the results to the entire researched community. From the perspective of the data collection method, it is worth noting that the research instrument was distributed online, and therefore, only individuals with Internet access could complete it. Another limitation refers to the subjectivity of the answers given and the possibility that they may have been influenced by recent experiences the respondents had in medical offices. From the perspective of the research variables, the study considered a limited number of variables. For this reason, future quantitative research should be conducted that studies this subject in greater detail, based on a more significant number of variables. Considering these limitations, it is necessary to perform more detailed and comprehensive quantitative studies using larger sample sizes. Additionally, it is recommended that qualitative research, such as focus groups and in-depth interviews, be conducted among medical professionals to determine the importance they attribute to the reputation of medical offices and their level of trust in patients.

## Conclusion

In the field of medical services, patient trust and reputation management are among the most crucial elements in the process of selecting a medical office. In ophthalmology and dentistry, reputation is vital to a medical practice’s success. This is because, at the level of these fields, the doctor-patient relationship directly involves both professional skills and a series of interpersonal interactions.

Patients’ trust in dental and ophthalmology medical offices is built based on several elements. Among the most critical factors are the patients’ previous experiences, the recommendations of relatives, online reviews, the online presence of the doctor and medical office, and the staff’s professionalism. A solid reputation in both fields studied plays a crucial role in attracting new patients and fostering the loyalty of existing ones. In this way, medical practices can enhance their market image. Recent technological developments have underscored the need for a proactive approach in reputation management. Thus, medical units had to increase their online visibility, communicate transparently, and respond effectively to all the reviews they receive. Considering all these aspects, it is worth noting that, in ophthalmology and dentistry, patient trust and reputation management are not merely auxiliary elements of medical offices but essential factors for their long-term success and sustainability.
